# Pleth variability index and respiratory system compliance to direct PEEP settings in mechanically ventilated patients, an exploratory study

**DOI:** 10.1186/s40064-016-3008-5

**Published:** 2016-08-20

**Authors:** Jing Zhou, Yi Han

**Affiliations:** Intensive Care Unit, Department of Geriatrics, Jiangsu Province Hospital, 300 Guangzhou Road, Nanjing, 210029 China

**Keywords:** Pleth variability index, Respiratory system compliance, Positive end-expiratory pressure, Perfusion index

## Abstract

**Objectives:**

To analyze the ability of pleth variability index (PVI) and respiratory system compliance (RSC) on evaluating the hemodynamic and respiratory effects of positive end expiratory pressure (PEEP), then to direct PEEP settings in mechanically ventilated critical patients.

**Methods:**

We studied 22 mechanically ventilated critical patients in the intensive care unit. Patients were monitored with classical monitor and a pulse co-oximeter, with pulse sensors attached to patients’ index fingers. Hemodynamic data [heart rate (HR), perfusion index (PI), PVI, central venous pressure (CVP), mean arterial pressure (MAP), peripheral blood oxygen saturation (SPO_2_), peripheral blood oxygen content (SPOC) and peripheral blood hemoglobin (SPHB)] as well as the respiratory data [respiratory rate (RR), tidal volume (VT), RSC and controlled airway pressure] were recorded for 15 min each at 3 different levels of PEEP (0, 5 and 10 cmH_2_O).

**Results:**

Different levels of PEEP (0, 5 and 10 cmH_2_O) had no obvious effect on RR, HR, MAP, SPO_2_ and SPOC. However, 10 cmH_2_O PEEP induced significant hemodynamic disturbances, including decreases of PI, and increases of both PVI and CVP. Meanwhile, 5 cmH_2_O PEEP induced no significant changes on hemodynamics such as CVP, PI and PVI, but improved the RSC.

**Conclusions:**

RSC and PVI may be useful in detecting the hemodynamic and respiratory effects of PEEP, thus may help clinicians individualize PEEP settings in mechanically ventilated patients.

## Background

Post end expiratory pressure (PEEP) reduces the collapse of alveoli during the expiratory phases due to its effect on functional residual capacity, which provides great support during mechanical ventilation in certain pathophysiological progress, such as severe pneumonia, atelectasis, ARDS, heart failure and pulmonary edema (Max et al. 1997). However, the benefits may come with certain adverse effects. Among them, hemodynamic disturbances drew a lot of attention from clinicians (Rajacich et al. 1988). Regarding to this issue, invasive measures such as Pulse Indicated Continuous Cardiac Output (Picco) monitoring has been introduced to help titrate PEEP in mechanically ventilated patients (Horster et al. 2012). But such invasive methods may aggravate patients’ affliction, with mechanical and infectious side effects, and proved not to change patients’ outcomes (Pavlovic et al. 2016). In this study we investigated the effects of increasing PEEP from 0 to 5 to 10 cmH_2_O on a novel parameter pleth variability index (PVI), a non-invasive hemodynamic indicator to help optimize PEEP settings in ventilated patients.

PVI has been proved to be a proper indicator in monitoring hemodynamic changes in mechanically ventilated patients, which is noninvasive, with good precision (Desebbe et al. 2010). It is calculated with the dynamic variations of perfusion index (PI) during respiratory cycle (DeBarros et al. 2015). Respiratory system compliance is the change in volume for any given applied pressure (Retamal et al. 2015). It represents the thoracic capacity to stretch and expand. This study was designed to explore the impact of PEEP on PVI and respiratory system compliance, elucidating how positive intra-thoracic pressure may affect hemodynamic and respiratory physiology, to help clinicians optimize PEEP settings during mechanical ventilation.

## Methods

The study protocol was ethically approved by the Institutional Review Board for human subjects. Written consent was obtained from patients’ legal family member before the research, as all patients were under sedation and mechanical ventilation.

This study totally enrolled 22 patients admitted in our ICU during the period from Dec 2014 to July 2015. Inclusion criteria included: age over 18 years old, admission in ICU during above period, severe illness in need of mechanical ventilation, and application of PEEP. Major diagnosis included the following: pulmonary infection, respiratory failure, internal hemorrhage with hepatic carcinoma, intra-cranial hemorrhage, post-operation of inguinal hernia, cerebral infarction, post-operation of hepatic transplantation and severe asthma. Exclusion criteria included: cardiac arrhythmias, intracardiac shunt, left ventricular dysfunction (ejection fraction <50 %), right ventricular dysfunction, unstable PI or PVI (defined as a variation in PI30 % over a 1-min period) (Desebbe et al. 2010), or any contraindication to the use of PEEP. Totally 4 patients were excluded because of unstable PI and undetectable PVI.

The study group consisted of 13 men and 5 women, aging from 47 to 98 years (mean: 78.2 ± 15.1 years). The object of the study was focused on the changing tendency instead of spot accuracy of PVI and PI. Patients with cardiac arrhythmia or inotropic or vasoactive reagents were excluded as the inclusion and exclusion criteria described above. The following results were proved to be effective with such patients as pulmonary infection, respiratory failure, internal hemorrhage with hepatic carcinoma, intra-cranial hemorrhage, post-operation of inguinal hernia, cerebral infarction, post-operation of hepatic transplantation and severe asthma.

PI and PVI were monitored with Masimo Set Radical-7 (Masimo Corp. Irvine, CA92618 USA), connected with a patently designed sticky pulse oximeter probe attached to the patients’ finger. Strong lights were avoided while monitoring the signal.

Other hemodynamic and respiratory variables such as mean arterial blood pressure (MAP), heart rate (HR), continuous central venous pressure (CVP), oxygen saturation (SpO_2_), oxygen content (SPOC), total hemoglobin (SPHB), respiratory rate (RR), expiratory tidal volume (VT) and respiratory system compliance (RSC) were monitored continuously with IntelliVue MP60 monitor, Philips, and Getinge respiratory ventilator, Maquet.

All patients were sedated and mechanically ventilated in a P-SIMV (Synchronized Intermittent Mandatory Ventilation) mode, allowing spontaneous breathes. We set up respiratory parameters to obtain 6–8 ml tidal volume per kg of body weight. All measurements were performed at 0, 5 and 10 cmH_2_O PEEP, each level of PEEP were stabilized for 15 min to obtain stable recordings, until the values’ curve reaching a plateau which lasted for 5 min at each level. Attention was given to maintain PVI and PI stable before and during data collection, to avoid deviation of value changes more than 15 %.

All data are presented as mean ± SD. Changes in respiratory and hemodynamic variables induced by different levels of PEEP were assessed with ANOVA. *P* value <0.05 was considered statistically significant. All statistical analyses were performed with PRISM 5 for Windows.

## Results

22 patients were enrolled. 4 patients were excluded for unstable or undetectable PVI. Mean Acute Physiology and Chronic Health Evaluation II (APACHE II) score of successfully enrolled patients were 23.8 ± 7.8. Hemodynamic and respiratory data at each step of the protocol for the 18 patients studied are shown in Table 1. Control pressure of mechanical ventilator were set at 13.2 ± 2.5 cmH_2_O to achieve tidal volume of 6–8 ml/kg body weight for each patient.

**Table 1:** Hemodynamic and respiratory data by different PEEP status

	PEEP (cmH_2_O)		
	0	5	10
HR (bpm)	87.9 ± 15.3	89.1 ± 16.1	89.3 ± 16.7
MAP (mmHg)	77.3 ± 10.8	78.8 ± 12.2	78.4 ± 12.3
SPO_2_ (%)	98.4 ± 2.0	98.7 ± 1.7	99.1 ± 1.3
SPOC (ml/dL)	14.5 ± 2.0	14.9 ± 1.9	14.9 ± 2.0
SPHB (g/L)	11.0 ± 1.6	11.3 ± 1.6	11.3 ± 1.6
RR (bpm)	20.4 ± 5.2	19.8 ± 4.9	21.2 ± 5.7

Data are expressed as mean ± SD

*PEEP* positive end-expiratory pressure, *HR* heart rate, *MAP* mean arterial blood pressure, *SPO*_2_ peripheral capillary oxygen saturation, *SPOC* peripheral oxygen content, *SPHB* peripheral hemoglobin, *RR* respiratory rate

### Changes in plethysmographic and hemodynamic data induced by different levels of PEEP

PEEP was set at 3 different levels (0, 5 and 10 cmH_2_O), hemodynamic changes were recorded at each level. All the whiskers bars in the figures represent SD.

**Fig. 1:**
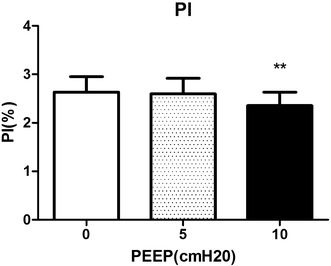
Changes of PI induced by different levels of PEEP (0, 5, 10 cmH_2_O). **Significant difference compared with group of PEEP at 0 cmH_2_O, *P* < 0.01 (0.0015)

**Fig. 2:**
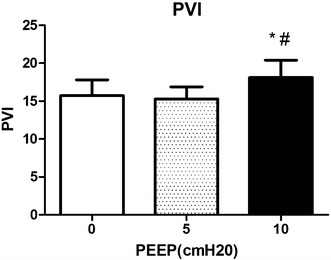
Changes of PVI induced by different levels of PEEP (0, 5, 10 cmH_2_O). *Significant difference compared with group of PEEP at 0 cmH_2_O, *P* < 0.01 (0.0070). ^#^Significant difference compared with group of PEEP at 5 cmH_2_O, *P* < 0.01 (0.0069)

**Fig. 3:**
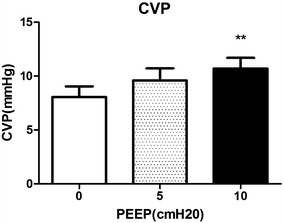
Changes of CVP induced by different levels of PEEP (0, 5, 10 cmH_2_O). **Significant difference compared with group of PEEP at 0 cmH_2_O, *P* < 0.01 (0.0004)

We observed remarkable decreases in PI at 10 cmH_2_O of PEEP compared with 0 cmH_2_O of PEEP (2.4 ± 1.2 compared with 2.6 ± 1.3, *P* = 0.0015), and 5 cmH_2_O of PEEP did not make great change of PI (2.6 ± 1.4) (Fig. 1). Meanwhile, 10 cmH_2_O of PEEP induced significant increases in PVI compared with 0 cmH_2_O of PEEP (18.1 ± 9.7 compared with 15.7 ± 8.9, *P* = 0.0070), while 5 cmH_2_O of PEEP did not make great change of PVI (15.3 ± 6.8) (Fig. 2). 10 cmH_2_O of PEEP induced significant increases in CVP compared with 0 cmH_2_O of PEEP, (9.6 ± 4.7 and 10.7 ± 4.1 compared with 8.1 ± 4.1, *P* = 0.0004), and such effects did not occur with 5 cmH_2_O of PEEP (Fig. 3).

### Changes in respiratory physiologic variables induced by different levels of PEEP

**Fig. 4:**
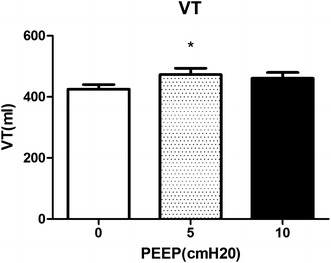
Changes of VT induced by different levels of PEEP (0, 5, 10 cmH_2_O). *Significant difference compared with group of PEEP at 0 cmH_2_O, *P* < 0.01 (0.0308)

**Fig. 5:**
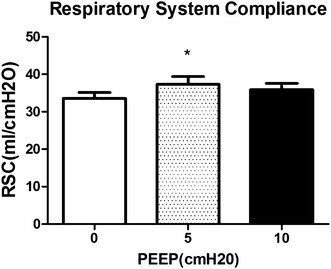
Changes of RSC induced by different levels of PEEP (0, 5, 10 cmH_2_O). *Significant difference compared with group of PEEP at 0 cmH_2_O, *P* < 0.05 (*P* = 0.0328; n = 18)

Only 5 cmH_2_O of PEEP improved VT and respiratory system compliance (RSC) (VT472.5 ± 90.2 and RSC 37.3 ± 8.6) compared with 0 cmH_2_O of PEEP (VT432.6 ± 56.6 and RSC 33.6 ± 6.6, *P* = 0.0308 and 0.0328). 10 cmH_2_O of PEEP neither contributed to tidal volume, nor to respiratory system compliance (VT460.9 ± 87.4 and RSC 35.9 ± 7.4) (Figs. 4, 5).

## Discussion

PVI is a newly invented noninvasive indicator to monitor hemodynamic variation. It is been proven PEEP settings could affect cardiac output in ventilated patients (Bruno et al. 2014). In our research, we demonstrated PEEP could increase CVP and PVI, thus inhibit both cardiac output and peripheral circulation. This conception has been widely established on large scale clinical researches, and exact cardiac output measuring requires an invasive technique such as Picco (Desebbe et al. 2010). Due to the prognosis non-improvement character of Picco, and the above established evidences, such invasive measurements were not recommended in this research.

Respiratory system compliance is directly affected by PEEP. The static compliance is calculated with the following formula: VT/(Pplat − PEEP). It represents respiratory system compliance during periods without gas flow (Laura et al. 2015). Clinically, appropriate PEEP would actually improve respiratory system compliance, but excessive PEEP may decrease the respiratory system compliance dramatically (Iaroshetskiĭ et al. 2014). Our research also demonstrated that 5 cmH_2_O PEEP improve respiratory system compliance, while 10 cmH_2_O PEEP did not have such effect, and may have the potential to decrease respiratory system compliance in ventilated patients.

For each critical patient, appropriate PEEP should be set to improve respiratory system compliance, then to achieve ideal tidal volume and oxygenation, and to avoid unexpected side effects of ventilation. Thus, individualized PEEP settings drew more and more attention from critical care clinicians (Iaroshetskiĭ et al. 2014; du Yun et al. 2014; El-Baradey and El-Shamaa 2014). Regard to individualizing PEEP settings, many researches have been conducted and certain indicators were generated to direct the procedure of ventilation (Gernoth et al. 2009). Such indicators are mostly invasive, with certain side effects. In our study, we investigated PEEP’s hemodynamic and respiratory effects, then to elucidate the possibility that PVI and respiratory system compliance may have the potentials to direct PEEP settings.

In this study, we demonstrated the relationships between PEEP and peripheral perfusion index PVI, PEEP and respiratory system compliance. Our results confirmed that certain levels of PEEP may improve respiratory system compliance thus induce a maximal tidal volume. But with the increasing levels of PEEP, systemic venous return and consequently cardiac output would be reduced (Kamath et al. 2010; Abdel-Hady et al. 2008), in the end peripheral perfusion would be decreased. Our aims were to find a balance among these factors, in search of an optional PEEP for each individual patient, with improvement of respiratory system compliance and least inhibitive cardiac effect. We detected the changes of PVI and CVP according to different levels of PEEP (0, 5, 10 cmH_2_O), and found out 5 cmH_2_O of PEEP induced a best respiratory system compliance thus an ideal tidal volume without affecting the circulation index including CVP and SpO_2_ values. As to how PEEP may improve the respiratory system compliance, most recordings agree that low levels of PEEP helps to reduce the collapse and to keep the opening of alveoli during the expiratory phases, increasing the functional capacity of lungs, thus providing great support with ventilated patients.

Clearly, various situations could affect the PVI, cardiac arrhythmias (for instance, atrial fibrillation), intracardiac shunt, left ventricular dysfunction (ejection fraction <50 %), and right ventricular dysfunction may affect the values of PI or PVI, because of insufficient or unstable cardiac output and thus an unstable peripheral saturation (Monnet et al. 2013; Loupec et al. 2011; Fu et al. 2012). Erroneously readings may be caused by hypoperfusion of the extremity, incorrect sensor application or highly calloused skin (Pi and Jin 2015). In regard to the possible effects of sympathetic nerves activities, all the patients were sedated to maintain an appropriate level of consciousness, with a Richmond Agitation-Sedation Scale of −2 to −3 to rule out such possible effects. All probes were carefully placed on the patients’ index finger with designed sticky patches.

It is been previously confirmed that ideal levels of PEEP may minimize the risk of ventilator-associated lung injury, thus reduce the duration of mechanical ventilation and ICU stay (Karsten et al. 2015). Out study introduced a non-invasive measure to help select an ideal PEEP to achieve better outcomes without causing severe side effects in cardiovascular system.

Our study had certain limitations. First, the results of this research shall be interpreted cautiously due to a relatively small size of samples. Due to certain contradictions of PVI monitoring, patients with cardiovascular shunt or other diseases affecting PVI/PI stability shall not be applied in this procedure for PEEP optimization purposes. Because of ethical issues, we were not allowed to set higher levels of PEEP in each patient, only 0, 5 and 10 cmH_2_O of PEEP were permitted. But these three levels of PEEP illustratively represented three different conditions, zero, mediate and relatively high levels of positive end intra-thoracic pressure. In our study, intermediate levels of PEEP were preferred. Though for each patient, clinician should combine PVI and respiratory system compliance together, carefully titrate PEEP into an optimization status.

In conclusion, in patients with mechanical ventilation, PVI and pulmonary compliance may be combined together to direct PEEP settings, to achieve optimal tidal volume, and to decrease inhibitive cardiovascular side effects.
